# 3D approaches to model the tumor microenvironment of pancreatic cancer

**DOI:** 10.7150/thno.42441

**Published:** 2020-04-06

**Authors:** Elena Tomás-Bort, Markus Kieler, Shreya Sharma, Juliana B Candido, Daniela Loessner

**Affiliations:** 1 Centre for Tumor Microenvironment, Barts Cancer Institute, Queen Mary University of London, EC1M 6BQ London, United Kingdom;; 2 Institute of Vascular Biology, Medical University of Vienna, 1090 Vienna, Austria;; 3 Department of Chemical Engineering and Department of Materials Science and Engineering, Faculty of Engineering, Monash University, Melbourne, VIC 3800, Australia;; 4 Department of Anatomy and Developmental Biology, Faculty of Medicine, Monash University, Melbourne, VIC 3800, Australia.

## Abstract

In tumor engineering, 3D approaches are used to model components of the tumor microenvironment and to test new treatments. Pancreatic cancers are a cancer of substantial unmet need and survival rates are lower compared to any other cancer. Bioengineering techniques are increasingly applied to understand the unique biology of pancreatic tumors and to design patient-specific models. Here we summarize how extracellular and cellular elements of the pancreatic tumor microenvironment and their interactions have been studied in 3D cell cultures. We review selected clinical trials, assess the benefits of therapies interfering with the tumor microenvironment and address their limitations and future perspectives.

## Introduction

Pancreatic cancer is one of the deadliest cancer and less than 9% of patients will survive for 5 years 1. Despite decades of research, this statistic has remained unchanged and emphasizes its substantial unmet need. Pancreatic cancer is predicted to be the second cause of cancer-related death by 2030 2. One reason for the high mortality of this disease is its asymptomatic early stages. Hence, the majority of patients are presented at the time of diagnoses at advanced stages, when the tumor has progressed and treatment options are very limited 3. In comparison to other malignant diseases, such as melanoma 4, lung cancer 5 and breast cancer 6, that have witnessed the implementation of targeted therapies or immunotherapies, systemic chemotherapy remains the standard treatment of metastatic pancreatic cancer. Advances have been made in our understanding of the genetic drivers of this disease. Yet, a therapeutic breakthrough in pancreatic cancer treatment is still missing 7.

Only 6.7% of oncology drugs in clinical development entering phase I achieve approval by the US Food and Drug Association 8. One reason for the lack of success of progressing from phase I is the inappropriate biodistribution and off-target toxicities observed with oncology drugs in patients. At the preclinical stage, 2D cell culture, animal and xenograft approaches are the most popular systems 9. However, these approaches fail to reflect the human tumor microenvironment (TME) and its molecular components accurately and can lead to non-translatable results 10, 11.

In recent years, the possibility of mimicking the TME *in vitro* by using tumor cells combined with a matrix or scaffold has helped researchers to narrow the uncertainty of the effectiveness of tested compounds 12. These TME *in vitro* models have been shown superior to 2D cell culture approaches, allowing 3D culture of multiple cell populations, cell-matrix interactions, treatment responses and the tumor heterogeneity as seen in patients diagnosed with pancreatic cancer 13-15.

**Figure 1:**
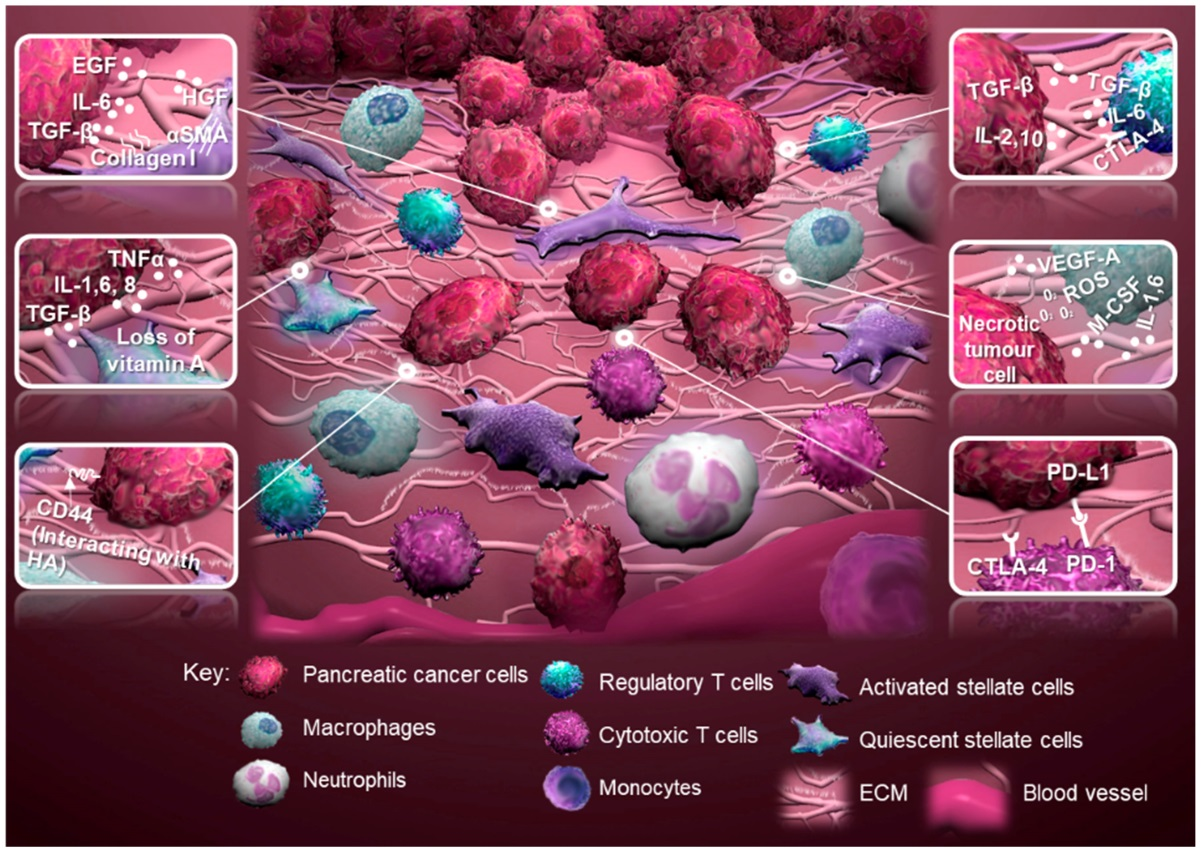
** Schematic of the tumor microenvironment of pancreatic cancer and tumor-stromal interactions.** Regulatory T cells produce an anti-inflammatory milieu through expression of cytotoxic T-lymphocyte antigen 4 (CTLA-4), while promoting tumor progression through transforming growth factor-beta (TGF-β). Activated stellate cells, characterized by alpha-smooth muscle actin (α-SMA) expression, contribute to tumor progression through multiple factors, for example collagen I, epidermal growth factor (EGF), hepatocyte growth factor (HGF) and expression of CD44 to interact with hyaluronic acid (HA). Quiescent stellate cells lose their capacity to store vitamin A caused by secretion of TGF-β, interleukin 1 (IL-1), IL-6, IL-8 and tumor necrosis factor (TNFα) by cancer and immune cells. Macrophages promote tumor progression through vascular endothelial growth factor-A (VEGF-A), IL-1, IL-6 and macrophage colony-stimulating factor (M-CSF), while increasing the mutational load of the tumor through the expression of reactive oxygen species (ROS). Cytotoxic T cells can be deprived from their tumoricidal activity by expression of CTLA-4 or programmed cell death protein 1 (PD-1; adopted from 19, 21, 22, 130). Abbreviation: ECM, extracellular matrix.

The most common type of pancreatic cancer is pancreatic ductal adenocarcinoma (PDAC) 16. PDAC tissues have a high stroma content, which accounts for up to 90% of the total tumor volume 17-20. Extracellular matrix (ECM) components, including collagen and hyaluronic acid (HA), and cellular components, including cancer-associated fibroblasts (CAFs) and immune cells, promote pancreatic cancer cell proliferation, immunosuppression and metastasis through a range of molecular factors 19, 21, 22 (**Figure 1**). These TME components form a fibrotic area that can block chemotherapeutics from reaching the tumor 23. The number of therapeutics targeting the pancreatic TME is steadily increasing. Therefore, there is a demand for preclinical models that can accurately mimic the TME as seen in patients 24-29 (**Table 1**).

## 3D cell culture approaches

**Figure 2:**
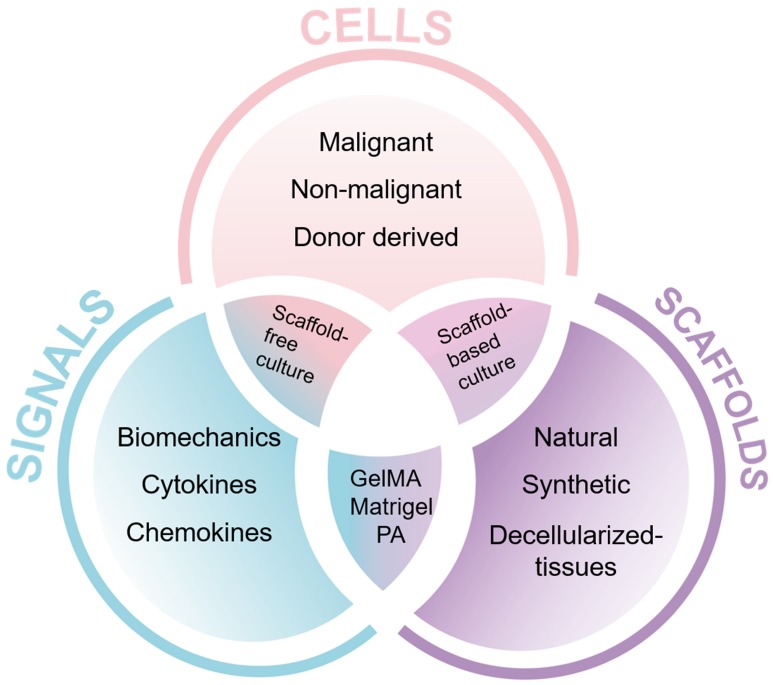
** Key components of 3D cell culture approaches.** The three major components to build a 3D cell culture model are cells, signals and scaffolds. Abbreviations: GelMA, gelatin methacryloyl 46; PA, peptide amphiphile 131.

The use of 3D cell cultures is an innovative approach that narrows the gap between traditional 2D cell cultures and animal models. It allows the control and manipulation of individual components of the TME in order to decipher their contribution to disease progression and treatment responses 30. Genetically engineered mouse models for PDAC, for example the LSL-Kras^G12D/+^, LSL-p53^R172H/+^, Pdx1-Cre (KPC) model, are resource-intensive and time-consuming 31. However, 3D cell cultures permit a validation step prior to animal testing, and when used in combination with animal studies reduce the number of animals needed 32. Immune cells play a role in tumor progression and response to treatment 33. 3D approaches allow the inclusion of human immune cells compared to patient-derived xenografts that are established in immunodeficient animals. With the possibility of humanized mouse models, xenograft approaches help us to assess the interaction of human immune cells with human tumors and circumvent difficulties which arise from interspecies differences 34, 35. When building a physiological mimic of a patient's tumor in the laboratory, the individual cell types 36, biomechanical and molecular signals and the type of scaffold need to be carefully considered (**Figure 2**).

**Figure 3:**
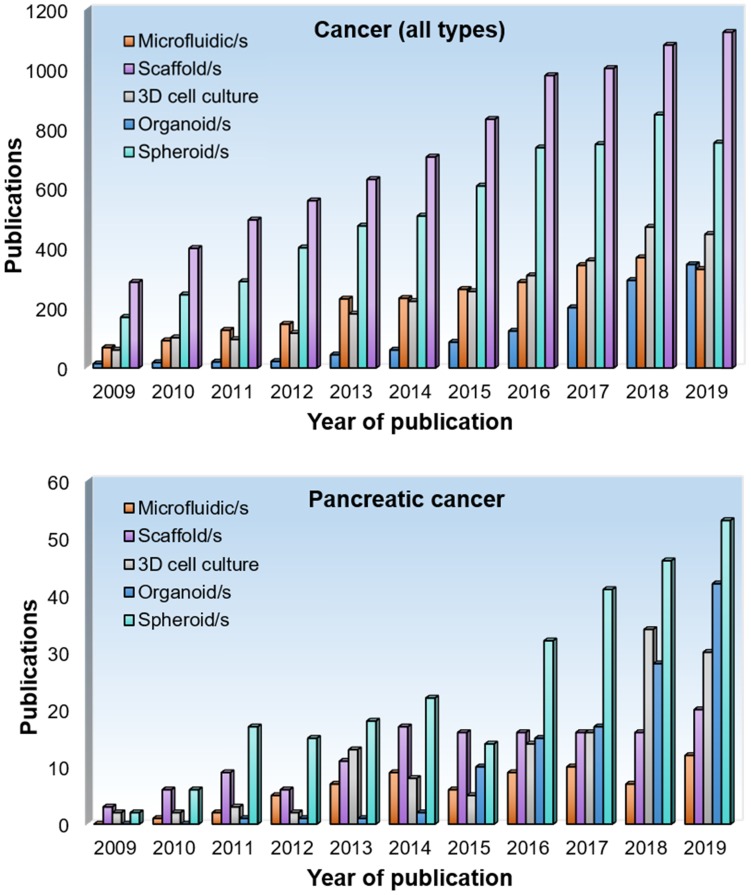
** Number of publications per year on 3D cell culture approaches in pancreatic cancer research.** A literature search was performed using PubMed using the following terms 'microfluidic/s', 'scaffold/s', '3D cell culture', 'organoid/s' and 'spheroid/s' in combination with 'cancer', 'tumor/tumor' or 'neoplasm' in the title or abstract. For the bottom graph, the word 'pancreatic' and 'pancreas' was included.

We conducted a literature research on different types of 3D cell culture methods used in cancer research (**Figure 3**). We found that scaffolds represent the most popular option in terms of the number of papers published per year according to PubMed containing the search terms 'scaffold(s)' and 'cancer', 'tumo(u)r' or 'neoplasm' in the title or abstract, accounting for 37% of our search results. For pancreatic cancer research, we found that spheroids are the most popular method, accounting for 42% of our search results. This is perhaps due to the relatively simple set-up of spheroid cultures and the low numbers of scaffold designs that are specific for this cancer compared to other cancer types. Other popular methods in pancreatic cancer research are organoids (33%), scaffolds (16%) and microfluidic systems (9%). Microfluidic devices arose in the early 2000's and are steadily gaining popularity in cancer research, accounting for 11% of our search results.

### Spheroids and organoids

Since the development of the hanging drop cell culture technique, spheroids have been used to study morphogenesis and the architecture and composition of malignant tissues 37. This method includes a coverslip or multi-well plastic lid to suspend cells in a drop of medium. The absence of direct contact with the surface forces cells to aggregate. This aggregation creates a cell cluster with limited oxygen diffusion in the center, leading to a hypoxic or apoptotic area that resembles the chemoresistant core of a tumor 38. Other traditional methods to grow spheroids from PDAC cells are non-adhesive plastic plates and spinner flask cultures 25. Bioengineering strategies, such as the use of methylcellulose 38, gelatin/ fibronectin multilayers 39, gelatin/polyvinyl alcohol scaffolds 40 or fibrous gelatin/polyglyconate scaffolds 41, delivered more uniform and reproducible methods.

These spheroid formation methods only partially provide PDAC cells with the characteristic dense stroma, tissue-like or cancer stem cell-like features found in patient tissues. To incorporate stromal components into tumor spheroids, non-malignant cells have been added to PDAC spheroids. Using a modified hanging drop method incorporating methylcellulose, the co-culture of PDAC cells with pancreatic stellate cells (PSCs) led to the production of a desmoplastic reaction, tumor-like cell morphology and tissue architecture 24. This model had high reproducibility and uniformity, however, spheroids were grown over a time frame of 7-10 days and had a low PSC seeding ratio, which needs to be considered when assessing treatment efficacy.

The surrounding matrix stiffness affects tumor spheroid formation and malignant behavior 42. Culture methods that allow spheroid growth with a physiological stiffness are crucial in modeling the TME properties of patient tissues. At early tumor stages, PDAC cells adhere on the basement membrane, which is mostly composed of type-IV collagen, fibronectin and laminin 37. Matrigel is a laminin-rich protein mixture derived from Engelbreth-Holm-Swarm mouse sarcoma and is the gold standard matrix for spheroid and organoid cultures. It has been used to mimic normal as well as diseased pancreatic tissue 43, 44. Because of its variable biological composition and animal-derived origin, Matrigel has limitations to truly recapitulate the physical and biochemical properties of the TME 45.

Alternatives include hydrogels formed from natural materials, such as collagen, fibrin or alginate, with improved tuneability and control over cell-matrix interactions. Semi-synthetic hydrogels that combine elements of synthetic and natural polymers, such as gelatin methacryloyl (GelMA) 46 or modified gelatin and HA 47, provide a higher control of the mechanical properties of the hydrogel while maintaining the biological cues to mimic cell-matrix interactions (**Figure 2**).

**Figure 4:**
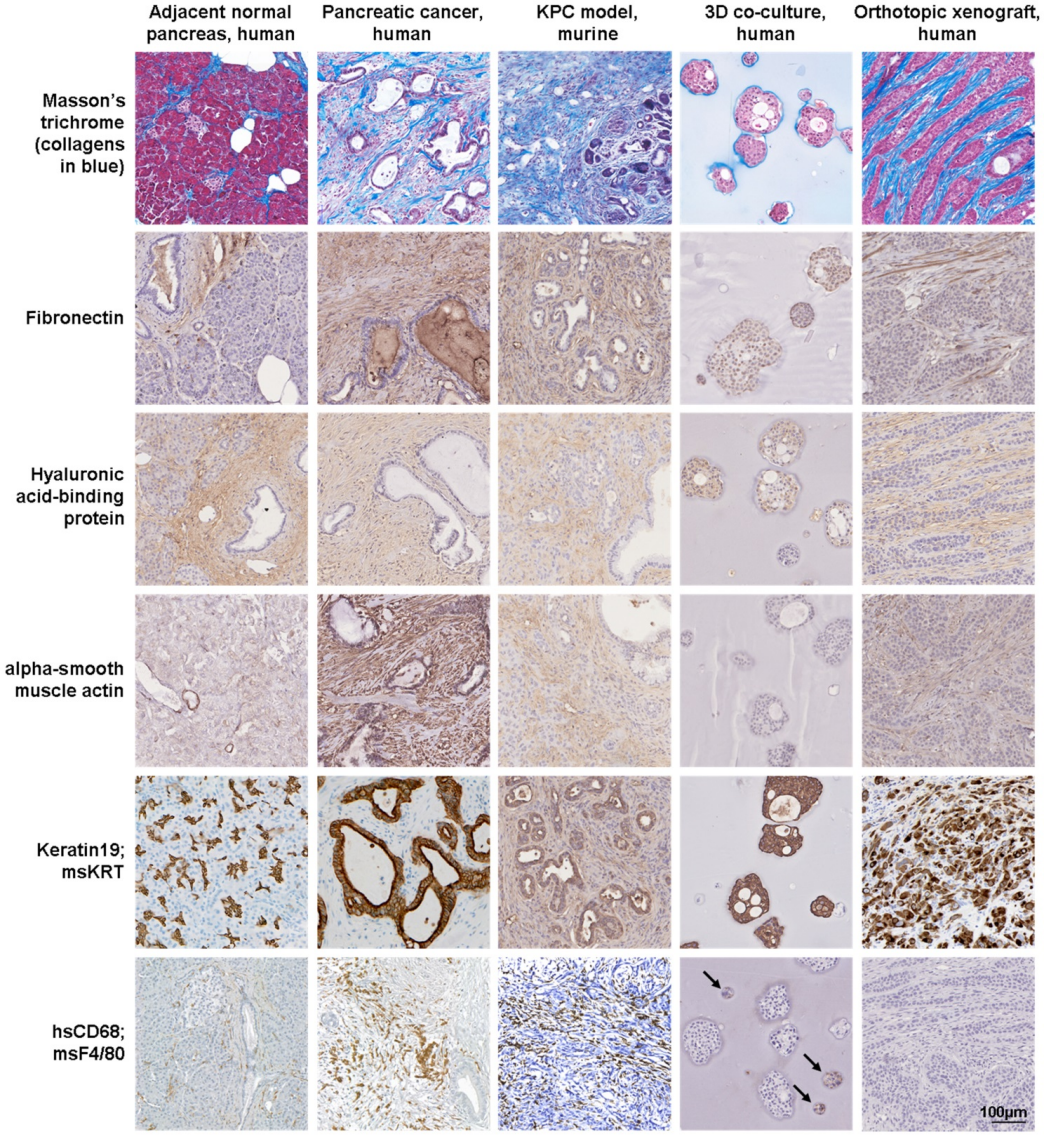
** Protein expression in pancreatic and xenograft tissues and 3D cell cultures.** Masson's trichome and hyaluronic acid-binding protein staining and immunoexpression of fibronectin, alpha-smooth muscle actin (fibroblasts), keratin19 (epithelial cells) and CD68 (macrophages) in normal pancreas and pancreatic cancer tissues, KPC tissues, 3D co-culture of pancreatic cancer cells with myeloid cells and orthotopic xenograft tissues. Human-specific (hs) antibodies were used for human-derived tissue, 3D co-culture (arrows) and xenograft tissue samples, while a mouse-specific (ms) pan-keratin (KRT) antibody and F4/80 were used for KPC tissue samples.

Although still rather ambiguous and disputed, the term organoid mostly refers to a cell cluster capable of self-renewal and self-organization 37. Organoids are established from normal or malignant tissue fragments, cell lines, embryonic or induced pluripotent stem cells, grown in 3D culture using an animal-derived ECM substitute, typically Matrigel or collagen gels, to produce an organ-like structure 37. Organoid cultures have been used to study human development and disease, as well as preclinical screening platforms for drug discovery and drug testing and as models of the TME 26, 43, 45. Spheroids are either self-assembling or are forced to grow as cell clusters or aggregates from a single cell suspension in the absence or presence of exogenous ECM components and recapitulate the 3D structure and organization of tissues or organs. The 3D co-culture of tumor spheroids with non-malignant cells, such as fibroblasts, endothelial or immune cells, has been used as preclinical platform for drug discovery and drug testing and as model of the TME 10, 24, 27, 38. Over the past decade, both 3D approaches have been increasingly used for pancreatic research (**Figure 3**). Our group uses tumor spheroids grown embedded within different hydrogel matrices, including collagen and semi-synthetic gels, for the 3D co-culture of PDAC cells with myeloid cells over 14-28 days and as an orthotopic xenograft approach to study the extracellular and cellular components of the pancreatic TME (**Figure 4**).

### Scaffold-based 3D cell culture

Scaffolds are materials with a 3D architecture of fibers and pores that act as the natural ECM structure where tumor and stromal cells reside, proliferate and migrate. Scaffold-based 3D cell cultures allow cells to grow in a 3D microenvironment without the need for cells to aggregate or form spheroids 30. With the use of new technologies, for example 3D bioprinting, different types of cells, materials and/or biological factors can be positioned with the scaffolding material in order to generate a tissue-specific TME 48. Although 3D bioprinting is still in its early stages, it has vast potential for drug discovery due to its spatiotemporal control and automation of 3D cell culture 49, 50. While 3D bioprinted scaffold-based models for pancreatic cancer have not yet been reported, 3D bioprinting has been used for example to produce models for cervical tumor 51, glioblastoma 52, breast cancer 53 and cancer metastasis 54. One of the challenges of 3D bioprinting is the development of bioinks. Bioinks are materials that are biocompatible and rapidly crosslinkable at ambient or body temperature without being cytotoxic 55.

Different types of scaffold materials have been used for 3D pancreatic cancer models, for example biocompatible materials, such as soft agar with a layer of Matrigel 56, polymeric scaffolds including a polyvinyl alcohol/gelatin mixture 40 and gelatin 25. The latter was used for the 3D co-culture of PDAC cells with CAFs, resulting in increased tumor cell proliferation and production of ECM components, such as collagen and glycosaminoglycans. Nonetheless, a major drawback of these scaffold-based cultures is the lack of vascularization, which is crucial for cancer metastasis.

### Microfluidic systems

Microfluidic 'organ-on-a-chip' approaches are used to maintain cells in 3D with the addition of perfused microchannels. This serves as a substitute for the vascular perfusion of the body required for the continuous supply of oxygen and nutrients and waste removal in an automated manner 57. However, the collection of cells at the end of experiments may be challenging and the fixed timescale design does not allow for scale-up cultures.

There are several microfluidic devices for PDAC using polydimethylsiloxane (PDMS) soft lithography 14, 27, 28. Hydrogels containing type-I collagen and HA were mixed with PDAC cells, fibroblasts or PSCs and loaded as a droplet in the inlets of the device. These 3D co-cultures were maintained for 3 days to allow cell-mediated ECM remodeling and to determine the cells' response to paclitaxel-based chemotherapy 14. In another study, a collagen-coated cyclic olefin polymer was used to culture PDAC cells, which showed an increased chemoresistance to cisplatin compared to spheroid cultures 15. To incorporate the physiological hemodynamics experienced by endothelial cells in the TME, a rotatory shearing motion was added. Endothelial cells were separated by a porous polycarbonate membrane from the 3D co-culture of PDAC cells and patient-derived PSCs. Transcriptome analysis revealed that the microfluidic 3D co-cultures were similar to the patient transcriptome 58. Using a different microfluidic system, pancreatic tumor spheroids were co-cultured with PSCs in a type-I collagen matrix. Each cell type was placed in a different channel separated by a porous PDMS membrane, allowing tumor cell migration towards the channel seeded with PSCs through the porous membrane 27.

It is well-known that cells in the TME sense the surrounding stiffness, which leads to the induction of an epithelial-to-mesenchymal transition (EMT) 59, 60. Therefore, it is possible that microfluidic cell culture systems based on PDMS and without the inclusion of cytocompatible materials or matrices may alter the behavior of PDAC cells, given that PDAC tissue has a stiffness of 4-8 kPa 60, 61. To study cell-matrix interactions, the properties and components of the ECM have to be systematically integrated into 3D approaches. We have provided an overview of the advantages and disadvantages of 3D approaches that incorporate key components of the pancreatic TME (**Table 1**). The different extracellular and cellular components of the pancreatic TME will be discussed in the following sections.

## Extracellular components of the pancreatic TME

The ECM of pancreatic tumors is composed of collagens, glycoproteins, proteoglycans, ECM regulators, ECM-affiliated proteins and secreted factors 62, which are released by stromal and tumor cells (**Figure 4**). The ECM not only contributes to the cellular architecture but also to homeostasis. In PDAC, ECM components impact tumor cell behavior, progression, metastasis, resistance to chemotherapy and are associated with poor clinical outcomes 63, 64.

### Collagen and promotion of a mesenchymal phenotype

Collagens are the main components of the PDAC-specific ECM 62. Type-I collagen is associated with enhanced signaling through the activation of focal adhesion kinase and Smad-interacting protein 1 and a loss of E-cadherin in PDAC cells, which are key factors during EMT 65, 66. High protein levels of type-I collagen were linked to shorter patient survival (6.4 months) compared to low levels (14.6 months), with expression found in primary PDAC tissues and metastatic lesions 67.

Due to the abundance of collagens in the ECM of PDAC and its implications in tumor biology, 3D cell cultures using collagen gels are widely used to study cell-matrix interactions (**Table 1**). In one study, type-I collagen was mixed with Matrigel by embedding PDAC cells into hydrogels with different collagen/Matrigel ratios to mimic the interstitial matrix and basement membrane. PDAC cells cultured in Matrigel exhibited an epithelial phenotype, while type-I collagen induced EMT and an invasive phenotype, which was due to an increase in matrix stiffness and fibril density 13. However, the highest stiffness tested was 1 kPa, which is not close to the reported PDAC tissue stiffness of 4-8 kPa 60, 61. One solution is to use collagen-like composite materials, for example GelMA, that permit a greater stiffness range 46.

In another study, the 3D culture of PDAC cells in a collagen matrix led to the upregulation of membrane type-I matrix metalloprotease (MT1- MMP), which was associated with resistance to the chemotherapeutic gemcitabine. Importantly, these effects were only seen in 3D not in 2D cell cultures, reinforcing the importance of 3D approaches for drug screening 23.

### Cell adhesion proteins and apoptosis resistance

Laminin is abundant in the basement membrane and mediates cell adhesion, while fibronectin is mainly in the interstitial matrix and secreted by PSCs to promote cell adhesion 19. In a simple study, plastic plates were coated with laminin or fibronectin. Both ECM proteins orchestrated resistance to apoptosis which occurred after cell detachment through cytochrome c-mediated caspase activation. However, the experiments did not include the fibrous collagen matrix of PDAC, in which laminin and fibronectin reside 68. One advantage of 3D models that integrate ECM proteins is that they facilitate long-term cell cultures. Fibronectin has been shown to maintain survival of T cells isolated from peripheral blood from patients with advanced cancer by stimulating them with anti-CD3 69. Laminin and fibronectin also limit early necrosis and apoptosis of PDAC cells 68. This may explain the correlation between high levels of fibronectin and a larger tumor size seen in patients 70.

### Hyaluronic acid as a tumor shield

HA is a non-sulphated glycosaminoglycan secreted by cancer cells and CAFs. PDAC is characterized by an accumulation of HA 71. Low molecular weight HA has been associated with aggressive and metastatic phenotypes through interaction with CD44, altered cell spreading as well as impaired vascularization and drug delivery 47, 72. High protein levels of HA were linked to shorter patient survival (9.3 months) compared to low levels (24.3 months), with expression found in primary PDAC tissues and metastatic lesions 67.

To improve drug diffusion, pegylated recombinant human PH20 hyaluronidase (PEGPH20) is being used to deplete HA enzymatically and is currently in clinical trials (**Table 2**). Studies in animal models showed an increase in survival when using a combination of PEGPH20 and gemcitabine compared to gemcitabine alone due to a normalization of the fluid pressure, reduced vascular collapse and increased permeability. This demonstrates the importance of HA in drug delivery as it may act as a shield that impedes chemotherapy delivery to tumor cells 72.

HA promotes mobility and drug resistance of tumor cells through the expression of hyaluronan-mediated motility (RHAMM) and interaction with CD44. The latter is a known stem cell marker and promotes metastasis through the loss of E-cadherin and accumulation of β-catenin. It also induces the expression of the transcription factor NANOG and stem cell regulators, which leads to the activation of the multidrug resistance protein 1 and chemoresistance in CD44-positive cells 73. Tumor cells expressing high levels of CD44 have been associated with gemcitabine resistance 74. RHAMM is overexpressed in poorly differentiated PDAC with high metastatic potential 75, indicating a role in epithelial transformation and a migratory phenotype.

A 3D system based on gelatin/HA hybrid hydrogels was developed that can be stiffened on demand using tyrosinase (**Table 1**). In this study, hydrogels of different stiffness, 1 kPa and 3 kPa, were prepared, which were then used to mimic the stiffness of normal and diseased pancreas tissues, respectively. Soft HA-containing hydrogels inhibited PDAC cell proliferation. In contrast, stiff HA-containing hydrogels promoted cell spreading and migration, which was attributed to EMT-induced changes 47. These results imply a synergic relationship between HA and stiffness in promoting a malignant cell behavior.

### Tumor-promoting effects of proteoglycans

Proteoglycans bind to different ECM components and influence protein activation and inhibition. One of the most commonly overexpressed proteoglycans in PDAC is Sparc/osteonectin, Cwcv and Kazal‐like domains proteoglycan (SPOCK1), which was characterized using co-cultured organoids. PDAC cells and fibroblasts were placed on top of mixed type-I collagen/Matrigel matrices using a 1:2 cell ratio. In response to transforming growth factor-beta (TGF-β), SPOCK1 had tumor-promoting effects by enhancing PDAC cell proliferation and modulation of collagen composition, which was not observed in 2D cell cultures 76.

Lumican is another proteoglycan found in PDAC and stromal tissues 77. In a retrospective study including PDAC patients, those with lumican-positive tumor cells survived longer than those with lumican- negative cells, whereas patients with lumican-positive stromal tissue had a lower survival than those with lumican-negative stroma. In contrast, when tumor cell monolayers and patient-derived xenografts were exposed to lumican they entered a quiescent state 77. This illustrates the complexity of PDAC-specific stromal proteins as lumican has both a tumor- suppressing and tumor-promoting role dependent on its location within the TME 78.

## Cellular components of the pancreatic TME

Previous research has identified key cellular components that are essential to the tumor biology of PDAC (**Figure 4**). The matrisome analysis of PDAC tumor tissues revealed that ECM proteins secreted by stromal cells are either positively or negatively correlated with patient survival 62. By using 3D approaches, the tumor-suppressing functions of stromal cells and therapeutic targets that interfere with their tumor-supporting properties can be explored by manipulating ECM proteins and cells.

### Cancer-associated fibroblasts

CAFs have a key role in PDAC development, progression and chemoresistance. They secrete multiple ECM components, forming a dense fibrous matrix characteristic of PDAC. CAFs are a mixed population of cells originating from resident fibroblasts, bone marrow-derived cells and PSCs. PSCs are the most studied fibroblast subtype in pancreatic cancer and have been used in 3D co-cultures with tumor cells 79.

Named after their star-like shape, PSCs are the key factors of the desmoplastic reaction in PDAC. In the normal pancreas, quiescent PSCs are found in the periacinar region and store vitamin A. Quiescent PSCs form only 4-7% of the total cell population 19. In PDAC, loss of vitamin A, triggered by cytokines secreted by tumor cells, chronically activates PSCs into their myofibroblast-like phenotype and modifies their star-like shape into a spindle shape. Activated PSCs consequently start expressing alpha-smooth muscle actin (α-SMA), type-I collagen, TGF-β and other proteins involved in cell proliferation, migration, ECM remodeling, EMT and inflammation 79.

In a microfluidic co-culture system PSCs were embedded in a type-I collagen matrix to study the distance at which tumor cells trigger their activation. PSC activation occurred over a distance of 1 mm away from tumor cells via secreted factors 27. Considering the heterogeneity of activated PSCs, the behavior of PSCs in proximity to tumor cells was investigated using co-cultured organoids in a 1:6 ratio of tumor cells to PSCs. The contact-dependent activation of PSCs by PDAC cells resulted in increased α-SMA levels, while paracrine activation led to increased interleukin 6 (IL-6) levels and other pro-inflammatory cytokines 20. Although PSCs contribute to the tumor biology of PDAC, targeting PSCs and reduction of the stromal compartment led to increased invasive tumor cell behavior in animal models of PDAC 80, 81. This is evidence of their tumor-modulating function. The use of 3D approaches helps us to understand why therapeutics fail and how to target specific subtypes of PSCs.

Multiple studies reported an increase in the metastatic phenotypes of PDAC cells when co-cultured with PSCs. Using a PDMS microchannel device, PDAC cells were co-cultured with PSCs as multicellular spheroids in a type-I collagen matrix (**Table 1**). 3D co-cultures promoted the alignment of collagen fibers and enhanced migration of both cell types. While PSCs exhibited F-actin stress fibers, which aligned with the collagen fibers and through activity of Rho-associated kinase, PDAC cells trailed PSCs along the collagen fibers 82. These findings suggest that PSCs assist tumor cells in their navigation outside the bulk tumor. PSCs also promote angiogenesis by assisting tumor cells to reach the blood vessels and metastasize to distant sites 83.

### Endothelial cells

PDAC is known for its poor vascularization which can cause a hypoxic microenvironment. Immature blood vessels are formed and require constant stimulation by the vascular endothelial growth factor (VEGF), which is secreted by PSCs 83. In contrast, blood vessel maturation was linked to better overall survival and cytotoxic immune cell infiltration in PDAC patients 84.

In the panstromal compartment (non-adjacent to tumor), which surrounds the juxta-tumoral stroma (<100 µm from tumor), a higher density of blood vessels has been observed 83. This increase in vascularization may be due to the lower endostatin concentration derived from the low number of PDAC cells and the high number of PSCs and macrophages that secrete VEGF to maintain endothelial cell survival. The expression of VEGF can also be triggered by a response to hypoxic conditions 85. In addition, the dense fibrous stroma and abundant HA content lead to a high interstitial pressure which causes compression of capillaries in PDAC tissues 85. In the juxta-tumoral stroma, the expression of endostatin, an angiogenic inhibitor derived from type-VIII collagen degradation, causes a hypovascular microenvironment 83. This is crucial when designing capillary-like networks in 3D cell cultures, as PDAC cells will inhibit vascular growth in a theoretical radius of 100 µm. Of note, PDAC cells and CAFs are known to secrete HA, which can also inhibit vessel formation 72.

The phenomenon of vascular inhibition has been studied using a microfluidic device, where two channels, one containing PDAC cells and the other one containing human umbilical vein endothelial cells (HUVECs), represent pancreatic ducts and blood vessels (**Table 1**). In proximity to PDAC cells, apoptotic endothelial cells appeared, which was attributed to the activin-ALK7 pathway and endothelial ablation 28.

PDAC cells, PSCs and HUVECs were co-cultured in a mixed type-I collagen/Matrigel matrix. When HUVECs were co-cultured with PDAC cells only, HUVECs survival and sprouting was decreased after 48-72 hours. In contrast, HUVECs co-cultured with activated PSCs formed luminal structures. These structures were suppressed when all-trans retinoic acid (ATRA)-induced quiescent PSCs were used instead of activated PSCs. These results demonstrate a pro- and anti-angiogenic role modulated by the activation of PSCs 83. ATRA is currently in clinical trials for PDAC and may be an effective stromal modulator used in combination with chemotherapy or immunotherapy (**Table 2**).

### Immune cells

Immunotherapy is achieving remarkable results in some solid tumors, including melanoma 4, lung cancer 5 and breast cancer 6. Because of the immunosuppressive microenvironment of PDAC and the immunological heterogeneity between patients, it is challenging to study and to treat patients with immunotherapies 86. Our understanding of the immune landscape of PDAC is constrained by the lack of appropriate experimental systems that can mimic the tumor immune microenvironment. 3D approaches hold great potential in providing tools to research the PDAC-specific immune system, even in a patient- specific manner.

#### Macrophages

In solid tumors, tumor-associated macrophages (TAMs) constitute the main population of immune cells. In the presence of apoptotic cells, macrophages secrete chemokines and cytokines assisting in the immune response. Monocytes differentiate into two simplified macrophage phenotypes: M1-like, associated with a pro-inflammatory and tumor-suppressing activity, or M2-like, linked to an anti-inflammatory and tumor-promoting activity 87.

3D cell culture models combining monocytes, fibroblasts and cancer cells have been valuable to understand monocyte differentiation into TAMs (**Table 1**). Tumor spheroids were co-cultured with fibroblasts and monocytes, leading to monocyte- derived macrophages with a M2-like phenotype. This was accompanied by the secretion of different cytokines, chemokines and growth factors by PDAC cells and fibroblasts. Cytokines like IL-6, IL-8 and IL-10 are known for their immunosuppressive role and involvement in M2 differentiation. The addition of macrophages did not influence the proliferation or survival of PDAC cells in the 3D co-cultures 87.

The concept of targeting TAMs arises from their role in angiogenesis, VEGF expression, ECM stiffening and suppression of T cells in PDAC. The influence of colony stimulating factor 1 receptor- positive (CSF1R+) macrophages in the pancreatic TME was studied in the KPC model. Inhibition of CSF1R showed a reduction in tumorigenesis and MYC gene programs. In addition, T cell genes were upregulated, promoting an adaptive immune response 88. This study demonstrated the profound effect of macrophages in the tumor immune microenvironment and their potential as a target in a clinical setting (**Table 2**).

#### Neutrophils

Neutrophils are some of the earliest immune cells recruited to the inflammatory TME and their accumulation is linked to poor prognosis 89. When tumor-associated neutrophils (TANs) phagocytize, they release reactive oxygen species and cytotoxic factors that impact the surrounding cells in the TME 89. Similarly to macrophages, neutrophils have a tumor-inhibitory N1-like phenotype and a pro-tumorigenic N2-like phenotype 90.

Spheroid cultures with HUVECs embedded in a type-I collagen matrix were used to study the pro-angiogenic effect of neutrophils. MMP9 added to spheroid cultures stimulated capillary-like networks. Neutrophils secreted MMP9 but not HUVECs or PDAC cells, the latter secreting VEGF instead. Antibodies against VEGF did not reduce the capillary-like networks of MMP9-exposed spheroid cultures, which demonstrates the need to study neutrophil-derived angiogenesis independent of VEGF 91. Neutrophils form neutrophil extracellular traps and expel their DNA, intracellular proteins and histones into the extracellular space 92. Neutrophil extracellular traps can also sequester circulating tumor cells and facilitate distant metastasis 93. These traps are important for inflammation and PDAC growth 92.

#### Tumor-infiltrating lymphocytes

A focus of immunotherapeutics has been tumor-infiltrating lymphocytes (TILs) in order to boost their cytotoxic effect through checkpoint blockades or with adoptive cell transfer. However, one major difficulty in PDAC is its immunosuppressive microenvironment that hampers the function of cytotoxic CD8+ T cells 94.

Activated PSCs have been associated with sequestration of CD8+ T cells in the panstromal compartment (non-adjacent to tumor), thus preventing T cell migration to the juxta-tumoral stroma (<100 µm from tumor) and isolating them from PDAC cells 95. This behavior was further investigated using 3D cell cultures. Organoids derived from patient-derived primary and metastatic tumor tissues and matched CAFs were grown in Matrigel. These organoid cultures were then placed in medium with suspended lymphocytes and analyzed for lymphocytic infiltration and migration towards the organoids. This tool may be used to validate immunotherapeutics aiming to improve lymphocyte infiltration into PDAC tissues 26.

The ability to study immunosuppressive targets in PDAC in a patient-specific manner is crucial for the improvement of immunotherapeutic responses. At present, several preclinical models are being used to screen combination strategies with chimeric antigen receptor (CAR) T cells and stroma-targeting therapies. However, these studies mostly use 2D cell cultures and animal models 96. Bioengineered 3D approaches can advance these studies by enabling real-time analysis of CAR T cell efficacy in a medium to high throughput manner, with tunable physical and biochemical properties and the inclusion of patient-derived immune cells 97.

## Conclusions and future perspective

In PDAC, the stroma accounts for most of the tumor volume, which is critical for tumor cell survival, proliferation and metastasis. Here we have discussed several 3D approaches that successfully incorporated key elements of the PDAC-specific TME into 3D cell cultures, including ECM constituents and non-malignant cell types. It is an exciting time for pancreatic cancer research, with the increase of stroma-targeting therapies and patient-specific 3D models that recreate a patient's unique TME. In spite of this, PDAC continues to be a cancer of substantial unmet need, and this is where 3D cell culture models can aid drug discovery and biological therapies.

**Figure 5:**
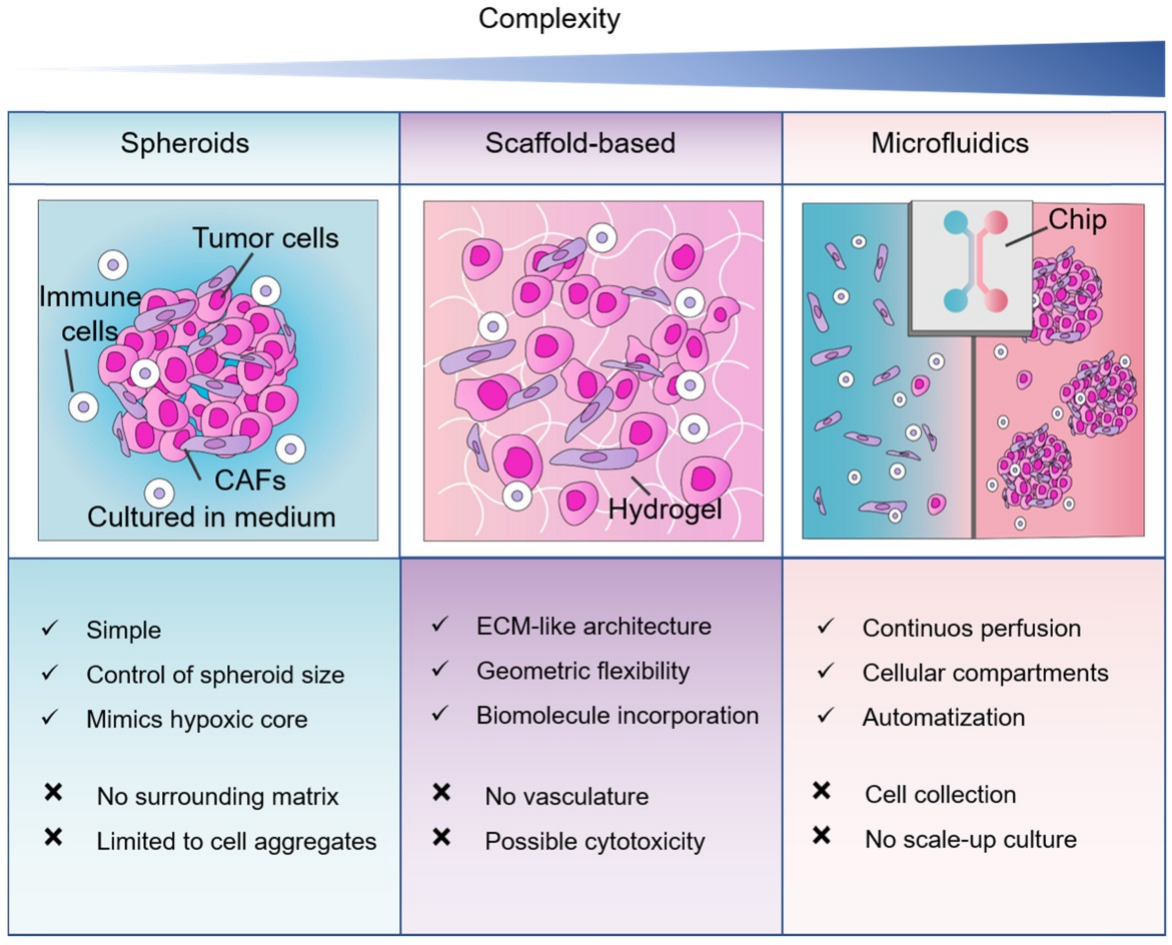
** Comparison between 3D cell culture approaches for co-culture of non-malignant and pancreatic cancer cells.** Three methods of 3D cell cultures, spheroids, scaffold-based and microfluidics in order of complexity. Abbreviations: CAFs, cancer-associated fibroblasts; ECM, extracellular matrix.

Our PubMed search results demonstrated that spheroids are the most popular 3D method in pancreatic cancer research. Depending on the terminology used, organoid cultures are as popular. In terms of mimicking tumor-stromal interactions, the literature reports a higher expression of ECM molecules in PDAC cells and CAFs in scaffold-based models in comparison to spheroids. Therefore, spheroid models may not entirely represent the ECM features of the pancreatic TME. However, the optimal method will always depend on the research question and the advantages and disadvantages of the 3D cell culture method considered (**Figure 5**).

The main advantage of bioengineered 3D approaches is their ability to provide a high degree of control and flexibility 30. The stiffness of the culture matrix can be precisely tuned by using semi-synthetic and synthetic hydrogels and varying their polymer concentration and crosslinking parameters 45. The stiffness of the PDAC-specific ECM is known to vary with time and space as well as with disease progression, which influences malignant and non-malignant cell behaviors. Nonetheless, a bioengineered 3D pancreatic cancer model with a physiologically relevant stiffness for both the extracellular and cellular components has yet to be developed. One possible reason for the lack of such a 3D approach may be that this area of research is dominated by tumor biologists and not by tissue engineers.

There are still many challenges to overcome in 3D approaches and to establish experimental models for translational research. For example, how to effectively mimic T cell behavior *in vitro* for 3D PDAC-specific immunotherapy assays or how to mimic the heterogenic vascular network of PDAC with epithelial cells to improve drug delivery. Maybe we can learn from the advances that have been made in 3D bioprinting human tissues. The emergence of 3D bioprinting is based on recent advances in material sciences and polymer chemistry. 3D bioprinting allows the generation of an artificial pancreas for the treatment of type 1 diabetes. Pancreatic islets are manufactured with supporting vascular and immune cells, biomimetic materials and bioactive factors for transplantation 98. Maybe we can also learn from the advances that have been made in regenerative medicine. Biological scaffolds can be derived from the ECM from decellularized and delipidized human pancreas tissues. After enzymatic digestions, the protein mixture forms a hydrogel allowing for 3D cell cultures 99. Biomimetic tissue engineering is a powerful approach to generate 3D cancer models. However, only a few scientists use these technologies. We found that most 3D cultures of human PDAC cells utilize soft reconstituted matrices that originate from murine tumors or tissues and contain undefined amounts of ECM proteins and growth factors. Other pitfalls of organoid cultures in Matrigel include a high batch-to-batch variation 100, overstimulation of cellular activity 101 and lack of tagging individual cell types for separate analysis after 3D culture 45, which pose risks for long-term cell expansion and controlled drug testing.

Another challenge is the design and analysis of cellular responses in 3D models and how we use them to understand the stromal response of a tumor and to measure the efficacy of stroma-targeting therapies. For example, model systems used to measure the interstitial fluid pressure and vascular collapse in pancreatic cancer have to be carefully considered depending on the research question asked and subsequent analysis. The physiology of fluid homeostasis in genetically engineered mouse models, xenograft and in vitro approaches, as well as pancreatic tumor tissues presents with different vascular features. Besides interstitial fluid pressure and hyaluronan, solid stress also contributes to the impaired perfusion in pancreatic cancer 12, 102. In terms of the complex epithelial-stromal interactions, maybe we can use 3D approaches to better understand the molecular mechanisms that are linked to the failure of some stroma-targeting therapies. For example, hedgehog inhibitors had dual activity in clinical trials with tumor-promoting and tumor-suppressing effects. Although hedgehog inhibitors had promising results at the preclinical stage, clinical studies failed to show a benefit and resulted in increased tumor growth and aggressiveness 81, 103.

Future bioengineered 3D approaches with control over patient-specific and biomechanical characteristics of the pancreatic TME have enormous potential to display features of desmoplasia and fibrosis that are important drivers of disease progression and immune escape mechanisms. Hybrid material approaches integrating fibrous scaffolds may recreate the matrix composition and architecture of primary tumor tissues and metastatic lesions. Modifications to these hybrid material approaches will make this new technology platform applicable to other stroma-rich cancers.

## Figures and Tables

**Table 1:** Selection of 3D approaches that incorporate elements of the tumor microenvironment of pancreatic cancer.

3D model	Element of the TME	Cell types	Advantage	Disadvantage	Reference
Hydrogel (collagen oligomer)	Collagen fibrillar structure	BxPC-3, PANC-1, MIAPaCa-2	Fibrillar structure used to simulate interstitial matrix	Physical properties, rather soft matrix	13
Hydrogel, 3D co-culture (collagen/HA, microfluidic culture)	Stroma: PSCs	PANC-1, neonatal human dermal fibroblasts, PSCs	Fusion of three channels into one to form tri-layer patterning of cells and matrix components	Limited to matrix components with compatible crosslinking methods, culture time limited	14
Spheroids (collagen coating, microfluidic culture)	Continuous perfusion	BxPC-3, PANC-1, MIAPaCa-2	24 cell culture regions per device	Dependent on cell spreading and adhesion to collagen	15
Organoids, 3D co-culture (Matrigel)	Stroma: PSCs, CAFs	Murine and patient- derived pancreatic cancer cells, PSCs, CAFs	Patient-derived multicellular 3D cultures, basement membrane mixture	Physical properties, rather soft matrix	20
Spheroids, 3D co-culture (modified hanging drop method)	Stroma: PSCs	AsPC-1, BxPC-3, Capan-1, PANC-1, MIAPaCa-2, PSCs	Reproducibility, uniformity	2:1 ratio of PDAC cells and PSCs, not representative of stroma content	24
Spheroids, 3D co-culture (gelatin porous microbeads, spinner flask culture)	Stroma: CAFs	PT45, NFs, CAFs	Microbeads provide a scaffold during microtissue formation and matrix production	Spheroid size is dependent on size of microbeads, cells need to be tagged and sorted prior to 3D co-culture for subsequent analysis	25
Organoids (Matrigel)	Stroma: CAFs, PBMCs	PANC-1, T cells, resected primary and metastatic tumor tissues, ascites, rapid autopsy specimen, murine xenografts, CAFs, PBMCs	Patient-derived multicellular 3D cultures, basement membrane mixture	Physical properties, rather soft matrix	26
Spheroids, hydrogel, 3D co-culture (type-I collagen, microfluidic culture)	Stroma: PSCs	AsPC-1, PANC-1, MIAPaCa-2, PSCs	Multichannel device with inter-channel cell migration and separation of different cell populations for subsequent analysis	Indirect 3D co-cultures, no direct cell-cell contacts, low cell numbers	27
Hydrogel, 3D co-culture (type-I collagen, microfluidic culture)	Blood vessel: HUVECs	BxPC-3, PANC-1, murine pancreatic cancer cells, HUVECs	Two-channel device with inter-channel cell invasion and separation of different cell populations for subsequent analysis, analysis of capillary-like networks	Physical properties, rather stiff matrix	28
Hydrogel (gelatin/HA)	Matrix stiffness	Colo-357	On-demand matrix stiffening and softening	Effects on remodeling induced by stromal cells, such as CAFs, not included	47
Hydrogel (polyacrylamide)	Matrix stiffness	AsPC-1, BxPC-3, Suit2-007	Control and reproducibility of mechanical properties with wide stiffness range (1-25 kPa)	Synthetic material, no biological cues provided	60
Spheroids, hydrogel, 3D co-culture (type-I collagen, microchannel device)	Stroma: PSCs	PANC-1, patient-derived PSCs	Visualization of collagen fibers and alignment	1:1 ratio of PDAC cells and PSCs, not representative of stroma content	82
Hydrogel, 3D co-culture (type-I collagen/Matrigel)	Stroma: PSCs Blood vessel: HUVECs	AsPC-1, Capan-1, Colo-357, primary PSCs, HUVECs	Organotypic multicellular 3D cultures, basement membrane mixture	Physical properties, rather soft matrix	83
Spheroids, 3D co-culture (poly-HEMA-coated multi-well dishes)	Stroma: CAFs, PBMCs	BxPC-3, HPAC, MIAPaCa-2, Pa-Tu 8902, fetal lung fibroblasts, CAFs, PBMCs	Reproducibility, uniformity	No consideration of physical properties and matrix components	87

Abbreviations: CAFs, cancer-associated fibroblasts; HA, hyaluronic acid; poly-HEMA, poly-2-hydroxyethyl methacrylate; HUVECs, human umbilical vein endothelial cells; NFs, normal fibroblasts; PBMCs, peripheral blood mononuclear cells; PDAC, pancreatic ductal adenocarcinoma; PSCs, pancreatic stellate cells.

**Table 2:** Selection of ongoing clinical trials that target the tumor microenvironment of pancreatic cancer.

Trial ID	Target(s)	Therapeutics	Phase	Mode of action	References
NCT02715804	ECM	PEGPH20 (HA degradation) + gemcitabine/ nab-paclitaxel	III	A major component of the ECM is HA, which raises the IFP within tumors and reduces drug delivery to malignant cells. PEGPH20 is a compound that degrades HA and normalizes IFP to enhance the delivery of cytotoxic agents.	85, 104
NCT02436668, completed	Immune cells	Ibrutinib (BTK inhibitor) + Gemcitabine /nab-paclitaxel	III	Bregs, mast cells and macrophages contribute to desmoplasia and an immunosuppressive TME. These three cell populations can be effectively targeted by BTK inhibitors like ibrutinib.	105
NCT02923921	Immune cells	AM0010 (activates T cells) + FOLFOX	III	AM0010 is a pegylated form of recombinant human IL-10. Preclinical studies showed that pegylated IL-10 has immunostimulatory effects that induce the activation, proliferation and survival of CD8+ T cells in the TME of PDAC.	106
NCT03126435	Tumor endothelial cells	EndoTAG-1 (liposome-embedded paclitaxel) + gemcitabine	III	Tumor endothelial cells lack the glycocalyx of the normal endothelium and therefore become negatively charged. This allows selective attachment and internalization of EndoTAG-1, which contains a positively charged lipid-based complex and leads to enhanced delivery of chemotherapeutic drugs.	107, 108
NCT03214250	Immune cells	APX005M (agonistic CD40 mAb) + gemcitabine/ nab-paclitaxel ± nivolumab (anti-PD-1 mAb)	II	CD40 is a costimulatory receptor and mainly found on antigen-presenting cells, in particular B lymphocytes, DCs and macrophages. Binding of CD40 ligands activates these cells, which have a crucial role in activating CTLs. In preclinical models, treatment with APX005M, an agonistic CD40 antibody, is associated with an influx of CTLs into tumors and subsequent tumor regression. A previous phase I study has shown immune activation and that the therapy was well tolerated.	109, 110
NCT02983578	Immune cells, PDAC cells, PSCs	AZD9150 (antisense STAT3) + durvalumab (anti-PD-L1 mAb)	II	The role of the transcription factor STAT3 is complex and it has diverse functions in different cell populations of the TME including PDAC cells and PSCs. Inhibition of STAT3 in preclinical models leads to reduced tumor growth and desmoplasia. There is conflicting evidence regarding the role of STAT3 inhibition in immune cells, particularly in the myeloid compartment.	111, 112
NCT02301130, completed	Immune cells	Mogamulizumab (anti-CCR4 mAb) + durvalumab (anti-PD-L1 mAb) or tremelimumab (anti-CTLA-4 mAb)	II	Tregs have a detrimental effect on anti-tumor immunity. These cells are attracted to the tumor by binding of ligands to CCR4. It has been shown that tremelimumab, an anti-CTLA-4 mAb, can eliminate Tregs in the TME, thus enhancing the effect of the CCR4-inhibitory antibody.	113
NCT03336216	Immune cells	Cabiralizumab (anti-CSF1R mAb) + nivolumab (anti-PD-1 mAb) or either investigator's choice chemotherapy	II	Inhibition of CSF1R signaling decreases the population of anti-inflammatory TAMs and furthermore functionally reprograms remaining macrophages to enhance antigen presentation and induce anti-tumor T cell responses in an animal model of PDAC. Investigations of this response revealed that CSF1R blockade also upregulates T cell checkpoint molecules, including PD-L1 and CTLA-4, thereby restraining beneficial therapeutic effects, which suggests a combination with checkpoint blockade.	88, 114
NCT02907099	Immune cells	BL-8040 (peptidic CXCR4 antagonist) + pembrolizumab (anti-PD-1 mAb)	II	Activated PSCs secrete CXCL12, a ligand for CXCR4. This attracts CD8+ T cells towards the juxta-tumoral stromal compartment and prevents their access to PDAC cells. In another study, FAP-positive stromal cells were identified as a source of CXCL12. Both studies reported that inhibition of the CXCR4-CXCL12 axis increases the number of intra-tumoral CTLs and improves anti-tumor responses.	95, 115, 116
NCT02758587	Immune cells, PDAC cells	Defactinib (FAK inhibitor) + pembrolizumab (anti-PD-1 mAb)	II	Signaling through the protein kinase FAK has been identified as a key pathway in PDAC cells regulating the fibrotic and immunosuppressive TME in PDAC. FAK inhibitors delayed tumor progression that was dependent on the presence of immune cells. A synergistic effect with anti-PD-1/PD-L1 therapy was observed in preclinical models.	117, 118
NCT03006302	Immune cells	Epacadostat (IDO inhibitor) + pembrolizumab (anti-PD-1 mAb) + CRS-207 ± GVAX and cyclophosphamide	II	IDO catalyzes the reaction from L-tryptophan to N-formylkynurenine and its overexpression in the TME leads to depletion of this amino acid. As L-tryptophan is essential for metabolic programming of T cells towards Th1 effector cells and natural killer cells functioning, IDO overexpression inhibits anti-tumor immune responses. In this trial, an IDO inhibitor is combined with an anti-PD-1 mAb, anti-cancer vaccines (CRS-207, GVAX) and a potent Treg depleting drug (cyclophosphamide).	119
NCT02210559	PSCs	FG-3019 (anti-CTGF mAb) +gemcitabine/ nab-paclitaxel	II	The pleiotropic matricellular signaling protein CTGF plays an important role in the development of desmoplasia by modulating integrin α5β1-dependent adhesion, cell migration, and type-I collagen synthesis. CTGF is overexpressed in PDAC cells and PSCs. Results from preclinical models suggest that the observed anti-neoplastic effect goes beyond enhanced drug delivery. The US Food and Drug Association has granted a fast track designation to pamrevlumab (FG-3019) for the treatment of patients with locally advanced, unresectable pancreatic cancer.	120-122
NCT03184870	Immune cells	BMS-813160 (CCR2/CCR5 antagonist) + nivolumab (anti-PD-1 mAb)	I/II	The G-protein coupled receptors CCR2 and CCR5 are expressed on the cell surface of monocytes and macrophages to stimulate their migration and infiltration into tumors. A preclinical study showed that dual targeting of CCR2+ TAMs and CXCR2+ TANs improves anti-tumor immunity and chemotherapeutic response in PDAC compared to either strategy alone.	123, 124
NCT02807844	Immune cells	Lacnotuzumab (anti-M-CSF-1 mAb) + spartalizumab (anti-PD-1 mAb)	I/II	TAMs mediate resistance to PD-1 inhibitors via upregulation of several anti-inflammatory mechanisms. These cells can be reduced by inhibiting the M-CSF-1 pathway with lacnotuzumab, a humanized anti-M-CSF-1 mAb, and spartalizumab, a humanized anti-PD-1 mAb, which may have synergistic anti-tumor activity.	114, 125
NCT03168139	Immune cells	Olaptesed pegol (CXCL12 inhibitor) ± pembrolizumab (anti-PD-1 mAb)	I/II	Olaptesed pegol blocks a key chemokine in the TME, CXCL12, which is involved in the homeostasis of blood and immune cells. In PDAC, CXCL12 acts as a communication point between tumor cells and the TME. In particular, it confers resistance to checkpoint inhibitors through T cell exclusion in preclinical models.	95, 116
NCT03307148	PSCs	ATRA + gemcitabine/ nab-paclitaxel	I	ATRA reduces the ability of PSCs to generate high traction forces, adapt to extracellular mechanical cues and force-mediated ECM remodeling which blocks PDAC cell invasion in 3D organotypic models.	126, 127
NCT02947165	Immune cells, PSCs	NIS793 (anti-TGF-β mAb) + PDR001 (anti-PD-1 mAb)	I	The robust desmoplastic reaction that accompanies PDAC progression is caused by TGF-β release from activated macrophages that stimulate PSCs to synthesize collagen type-I and fibronectin. Furthermore, TGF-β attenuates tumor response to PD-L1 blockade by contributing to exclusion of T cells. Synergistic effects of blocking these two pathways have shown promising preclinical results.	128, 129

Abbreviations: AM0010, pegylated human IL-10; ATRA, all-trans retinoic acid; Bregs, B regulatory cells; BTK, Bruton's tyrosine kinase; CCR2, C-C chemokine receptor 2; CCR4, C-C chemokine receptor 4; CCR5, C-C chemokine receptor 5; CD40, cluster of differentiation 40; CSF1R, colony stimulating factor 1 receptor; CTGF, connective tissue growth factor; CTLA-4, cytotoxic T-lymphocyte antigen 4; CTLs, cytotoxic T-lymphocytes; CXCL12, C-X-C chemokine ligand 12; CXCR2, C-X-C chemokine receptor 2; CXCR4, C-X-C chemokine receptor 4; DCs, dendritic cells; ECM, extracellular matrix; FAK, focal adhesion kinase; FAP, fibroblast activation protein; HA, hyaluronic acid; IDO, indoleamine 2,3-dioxygenase; IL-10, interleukin 10; IFP, interstitial fluid pressure; mAb, monoclonal antibody; nab-paclitaxel, nanoparticle albumin-bound paclitaxel; M-CSF1, macrophage colony-stimulating factor 1; PD-1, programmed cell death protein 1; PD-L1, programmed cell death-ligand 1; PDAC, pancreatic ductal adenocarcinoma; PEG, polyethylene glycol; PEGPH20, pegylated recombinant human PH20 hyaluronidase; PSCs, pancreatic stellate cells; STAT3, signal transducer and activator of transcription 3; TAMs, tumor-associated macrophages; TAN, tumor-associated neutrophils; TGF-β, transforming growth factor-beta; Th1, T helper 1; TME, tumor microenvironment; Tregs, T-regulatory cells.

## Abbreviations

α-SMA: alpha smooth muscle actin
ATRA: all-trans retinoic acid
CAFs: cancer-associated fibroblasts
CAR: chimeric antigen receptor
CSF1R: colony stimulating factor 1 receptor
ECM: extracellular matrix
EGF: epidermal growth factor
EMT: epithelial-to-mesenchymal transition
GelMA: gelatin methacryloyl
HA: hyaluronic acid
HUVECs: human umbilical vein endothelial cells
IL-6: interleukin 6
KPC: LSL-Kras^G12D/+^: LSL-p53^R172H/+^: Pdx1-Cre
MT1-MMP: membrane type-I matrix metalloprotease
PDAC: pancreatic ductal adenocarcinoma
PDMS: polydimethylsiloxane
PEGPH20: pegylated recombinant human PH20 hyaluronidase
RHAMM: receptor for hyaluronan-mediated motility
PSCs: pancreatic stellate cells
SPOCK1: Sparc/osteonectin: Cwcv and Kazal‐like domains proteoglycan
TAMs: tumor-associated macrophages
TANs: tumor-associated neutrophils
TGF-β: transforming growth factor-beta
TILs: tumor-infiltrating lymphocytes
TME: tumor microenvironment
VEGF: vascular endothelial growth factor
